# Local persistence of novel regional variants of La Crosse virus in the Northeast USA

**DOI:** 10.1186/s13071-020-04440-4

**Published:** 2020-11-11

**Authors:** Gillian Eastwood, John J. Shepard, Michael J. Misencik, Theodore G. Andreadis, Philip M. Armstrong

**Affiliations:** 1 Center for Vector Biology & Zoonotic Diseases, Department of Environmental Sciences, The Connecticut Agricultural Experiment Station, New Haven, Connecticut USA; 2 Present Address: Department of Entomology, Virginia Polytechnic Institute & State University, Blacksburg, Virginia USA

**Keywords:** Arbovirus, Vector, Mosquito species, La Crosse virus, Pathogen persistence, Genetic distinction, Public health risk

## Abstract

**Background:**

La Crosse virus (LACV) (genus *Orthobunyavirus*, family *Peribunyaviridae*) is a mosquito-borne virus that causes pediatric encephalitis and accounts for 50–150 human cases annually in the USA. Human cases occur primarily in the Midwest and Appalachian regions whereas documented human cases occur very rarely in the northeastern USA.

**Methods:**

Following detection of a LACV isolate from a field-collected mosquito in Connecticut during 2005, we evaluated the prevalence of LACV infection in local mosquito populations and genetically characterized virus isolates to determine whether the virus is maintained focally in this region.

**Results:**

During 2018, we detected LACV in multiple species of mosquitoes, including those not previously associated with the virus. We also evaluated the phylogenetic relationship of LACV strains isolated from 2005–2018 in Connecticut and found that they formed a genetically homogeneous clade that was most similar to strains from New York State.

**Conclusion:**

Our analysis argues for local isolation and long-term persistence of a genetically distinct lineage of LACV within this region. We highlight the need to determine more about the phenotypic behavior of these isolates, and whether this virus lineage poses a threat to public health.
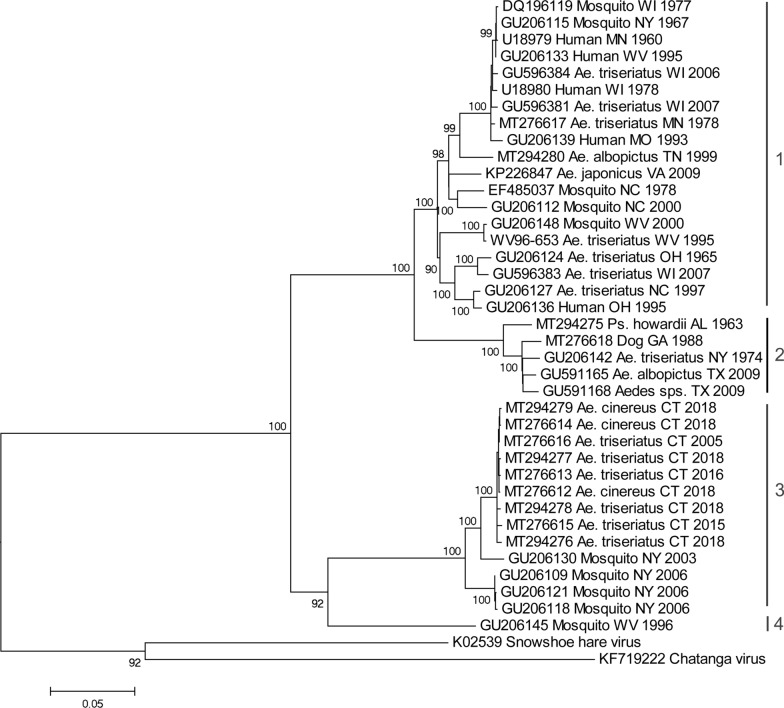

## Background

La Crosse virus (LACV) (genus *Orthobunyavirus*, family *Peribunyaviridae*) is a mosquito-borne virus, associated with clinical cases of pediatric encephalitis concentrated in the Midwest and Appalachian regions where it is also detected in mosquitoes during surveillance activities. Symptoms of La Crosse encephalitis include headache, fever, vomiting, and disorientation which may lead to seizure, coma, and death in severe cases [1]. Notable clusters of illness have occurred in Ohio, Wisconsin, Tennessee, and North Carolina with a substantial socioeconomic burden associated [1–4]. In contrast, only one locally-acquired case of LACV has been reported in the northeastern USA during the last 20 years, occurring in upstate New York in 2010 and was non-neuroinvasive [5]. In addition, two human cases were reported in Rhode Island, during 2018 and 2019, but both were imported as these individuals had a travel history outside that state (Rhode Island DPH, personal communication). LACV was first detected in the New England region in a pool of mosquitoes collected in Connecticut in 2005 during statewide surveillance for arboviral pathogens [6]. Phylogenetic analysis indicated that the Connecticut isolate represented a genetically distinct lineage that diverged earliest from viruses circulating in other geographic regions of the USA [6, 7].

Despite the disease being associated within the Midwest and Appalachian regions of the USA, LACV may represent an unrecognized public health risk to residents of New England and New York. There is a need to understand the transmission cycles, prevalence, and strains of LACV in this region. *Aedes triseriatus* serves as the main vector of LACV which is broadly distributed throughout the eastern half of the USA [8]. Nevertheless, this species is likely under-sampled during statewide surveillance efforts because it does not readily enter standard CDC light traps or gravid traps which are routinely used [3, 9]. Additional sampling and testing of *Ae. triseriatus* and other locally abundant mosquito species are needed to accurately estimate the entomological risk of LACV in enzootic sites.

Here we report the results of mosquito monitoring activities detecting LACV in Connecticut. In 2018, we conducted two surveys that intensified the extent of mosquito sampling and testing in central and southwestern Connecticut and highlighted increased detection of LACV activity [10]. In addition, routine statewide surveillance in Connecticut, ongoing since 1997, detected four additional isolates of LACV since 2005. We present evidence for ongoing circulation of LACV in this region, and report on the entomological and viral phylogenetic data that supports local persistence of LACV in Connecticut.

## Methods

### Entomological data

#### Mosquito collections

**Fig. 1:**
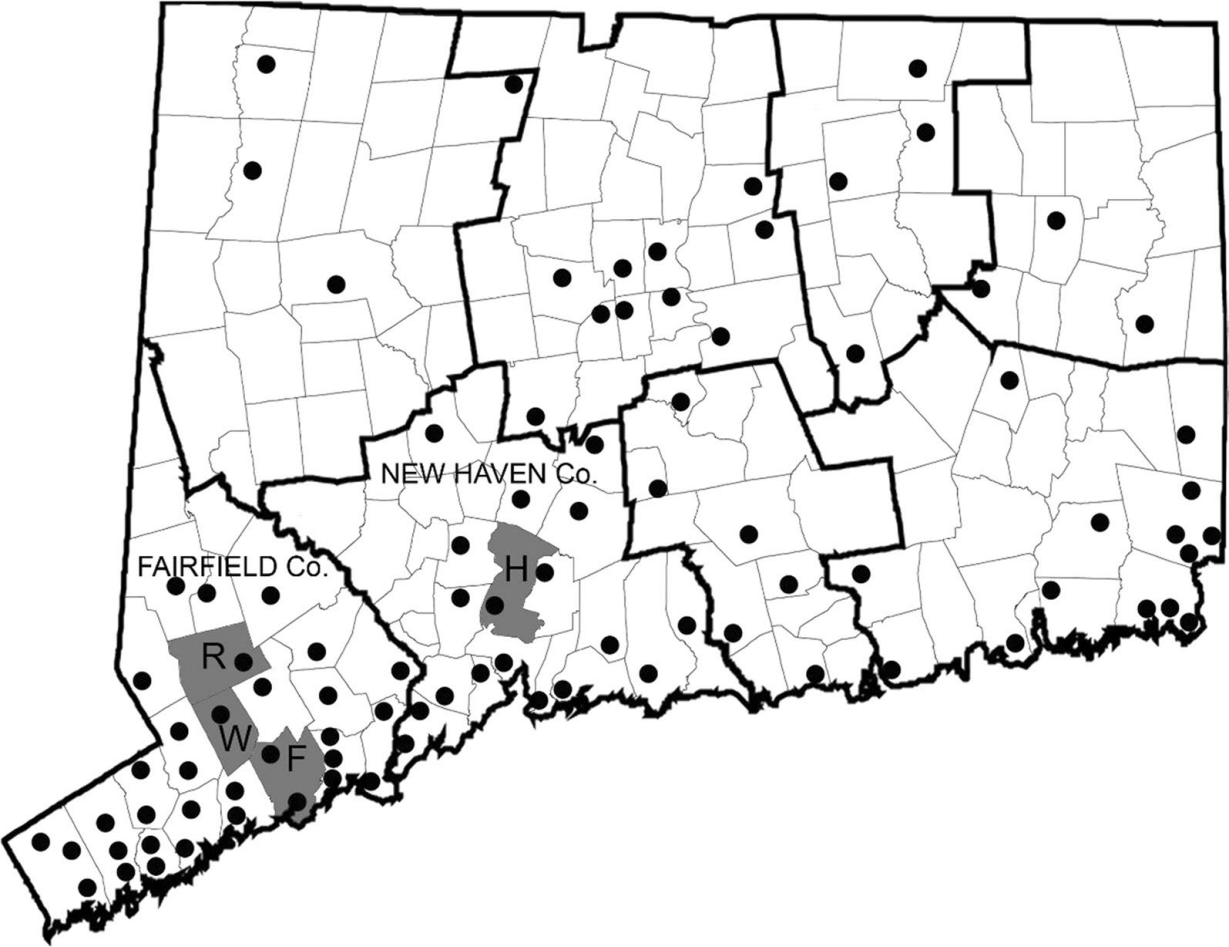
Map of Connecticut showing town and county boundary lines and location of mosquito trap sites (shown as black circles) from the statewide surveillance programme (survey i), the lure evaluation (survey ii), and focused LACV-sampling (survey iii). Towns where LACV-positive mosquito pools were collected are shaded in grey. *Abbreviations*: R, Redding; W, Weston; F, Fairfield; H, Hamden
(ii) A trapping-lure comparison study (reported by Eastwood et al. [10]) was conducted in Stamford, CT and Hamden, CT, capturing 33,649 and 14,085 individuals, respectively, using BG-Sentinel traps baited with CO_2_ and different chemical lures. A total of 47,734 mosquitoes (32 species of 8 genera) were trapped and identified over 27 days of sampling in July and August 2018, and pooled for virus detection.
(iii) Thirdly, focused sampling was performed during 2017 and 2018 at the location where LACV was first detected in the region, (Fairfield, CT; 41.19467, -73.32730), as well as two nearby sites: Easton, CT (41.28032, -73.30308) and Redding, CT (41.30972, -73.32178). Here, CDC light traps, BG-Sentinel traps, MMX traps, and gravid traps, as well as GAT traps (Biogents) were deployed overnight at sites for two consecutive nights each week, August-October 2017 and May-October 2018 targeting adult mosquitoes. In addition, as a pilot study of vertical transmission at those sites, six oviposition cups (black plastic casino cups, lined with seed germination paper), were placed in the field and checked weekly to collect container-breeding mosquito eggs.

Isolates of LACV analyzed in the present investigation were obtained from field-collected mosquitoes that were procured from the following:

(i) Connecticut state has conducted annual monitoring of mosquitoes at 36 locations since 1997 and was increased to a total of 91 locations (8 counties) since 2001. Field surveillance was conducted using CDC light traps and gravid traps, and at some sites, BG-Sentinel traps (Biogents, Regensburg, Germany), followed by mosquito identification and viral testing across the eight counties of Connecticut. Each site was sampled at least once every 10 days from June-October (Fig. 1). We consider here, data from the 2005–2018 surveillance seasons.

Mosquitoes captured within each study were sorted from other insect fauna, identified morphologically to species level using a key [11], with a cold-chain maintained throughout.

#### Virus detection

Adult mosquitoes were identified and screened for arbovirus as follows. Pools of up to 50 mosquitoes (grouped by species, location and capture date) were homogenized and inoculated on a Vero (African Green monkey kidney) cell line, for evidence of arboviral infection, as described Eastwood et al. [10]. Briefly, mosquitoes were homogenized in a vial with 1 ml PBS-G (phosphate-buffered saline containing 0.5% gelatin, 30% rabbit serum and 1× antibiotic/antimycotic) and a copper BB pellet, using a mixer-mill set for 4 min at 25 cycles/second. Samples were then centrifuged for 5 min at 7000× *rpm* at 4 °C. The supernatant (100 μl) was inoculated onto a confluent monolayer of Vero cells in 25-cm^2^ culture flasks, allowed to absorb for 5 min on a plate rocker, then provided with 4 ml of minimum essential media supplemented with 10% fetal bovine serum, 1× antibiotic/antimycotic. Flasks were incubated at 37 °C with 5% CO_2_ and examined daily for cytopathic effect (CPE) for up to 7 days. Infected cell cultures showing CPE were harvested and stored at -80 °C. To identify viruses, RNA was extracted from isolates using a QIAampViral RNA mini kit (Qiagen, Germantown, MD, USA), eluted in a final volume of 70 μl. A reverse transcription polymerase chain reaction (RT-PCR) was performed in a 25 μl reaction using a Titan one-tube RT-PCR system (Roche Diagnostics, Indianapolis, IN, USA) with generic orthobunyavirus primers [10, 12]. Amplification products of the appropriate size, were purified as per Eastwood et al. [10], then commercially sequenced (Science Hill DNA Analysis Facility, Yale University, New Haven, CT, USA).

Mosquito eggs collected using the oviposition cups, as part of the third collection study, were reared to adults in the laboratory of the CAES at 25 °C with a 16:8 h light:dark photoperiod. This F_1_ generation was screened for virus as above, to test for evidence of LACV having been vertically transmitted.

Minimum infection rates [MIR] of LACV were determined parsed by mosquito species/site/year using the CDC-provided Excel Add-in tool for calculating bias-corrected maximum likelihood estimate pooled infection rates [13].

### Phylogenetic data

#### Nucleotide sequencing and genetic analyses

Viral RNA was isolated from virus cultures using the QIAamp viral RNA Kit (Qiagen, Valencia, CA, USA). RT-PCR was performed using the Titan One-Tube RT-PCR System (Roche Diagnostics) with primers targeting each of the three genomic segments of LACV. Primer pairs BUNS+new/BUNS-new and M14C/M4510r were used to amplify the entire S and M segments as previously described (Armstrong & Andreadis [6]). In addition, a portion of L segment was amplified using primers LACL2fwd (5’-GTA GTG TAC TCC TAT CTA CAA AAC-3’) and LACL1077rev (5’-GTT GAT ATA CCC TTT ATG CTT TG-3’) [6]. Amplification products were purified using the PCR purification kit (Qiagen) and sequenced at the DNA analysis facility using an Applied Biosystems 3730xl 96-capillary genetic analyzer (Applied Biosystems, Foster City, CA, USA). Overlapping sequence chromatograms were aligned and edited using ChromasPro (Technelysium Ltd., Tewantin, Australia) and virus sequences were deposited in GenBank (Additional file 1: Table S1).

Edited nucleotide sequences were compared to those available on GenBank using the BLASTn search algorithm (http://blast.ncbi.nlm.nih.gov/Blast.cgi). Multiple sequence alignments were generated by the ClustalW algorithm and phylogenetic relationships were evaluated by maximum-likelihood analysis in MEGA 6.0. The optimal nucleotide substitution was selected by performing ML fits of 24 different models in MEGA. Support for individual nodes was evaluated by performing 1000 bootstrap replicates.

## Results

### Entomological data

A total of 14 new isolates of LACV were obtained during the mosquito surveillance activities in CT: (i) routine arbovirus monitoring detected additional isolates of LACV from the town of Redding in both 2015 and 2016, and two isolates in the towns of Weston and Fairfield during 2018, all within Fairfield county (Fig. 1, Table 1); (ii) of 14,085 mosquitoes captured at the Hamden location during the Lure evaluation study in July and August 2018, 4 novel isolates of LACV were made from three different mosquito species (Table 1); (iii) focused LACV surveillance extension in Fairfield county captured a total of 16,325 mosquitoes at three different sites, which were screened as 310 pools. Six isolates of LACV were identified at the Fairfield and Redding sites between 10th July and 9th October, during this intensive capture study. These isolates came from pools of *Ae. triseriatus* with the exception of one isolate from *Ae. cinereus* (Table 1).

**Table 1:** Source of LACV isolates in Connecticut

Year	Date	Town	Trap type	Species	Pool size
Survey 1: Connecticut-wide annual surveillance (2005–2018)					
2005	15-Aug-05	Fairfield	Light trap + CO_2_	*Aedes triseriatus*	4
2015	28-Jul-15	Redding	Light trap + CO_2_	*Aedes triseriatus*	17
2016	22-Sep-16	Redding	Light trap + CO_2_	*Aedes triseriatus*	1
2018	8-Aug-18	Weston	Light trap + CO_2_	*Aedes cinereus*	19
2018	13-Sep-18	Fairfield	Light trap + CO_2_	*Aedes cinereus*	6
Survey 2: Lure evaluation study (Hamden and Stamford; July-August 2018)					
2018	1-Aug-18	Hamden	BG-Sentinel + CO_2_	*Aedes canadensis*	50
2018	24-Jul-18	Hamden	BG Sentinel + CO_2_	*Aedes triseriatus*	44
2018	24-Jul-18	Hamden	BG Sentinel + CO_2_	*Aedes trivittatus*	40
2018	6-Aug-18	Hamden	BG Sentinel + CO_2_	*Aedes triseriatus*	14
Survey 3: Focused LACV surveillance in Fairfield county (2017–2018)					
2018	10-Jul-18	Fairfield	Light trap	*Aedes cinereus*	32
2018	11-Jul-18	Fairfield	BG Sentinel + CO_2_	*Aedes triseriatus*	3
2018	11-Sep-18	Redding	BG Sentinel + CO_2_	*Aedes triseriatus*	12
2018	20-Sep-18	Redding	BG Sentinel + CO_2_	*Aedes triseriatus*	3
2018	2-Oct-18	Redding	BG Sentinel + CO_2_	*Aedes triseriatus*	1
2018	9-Oct-18	Redding	BG Sentinel + CO_2_	*Aedes triseriatus*	8

The minimum infection rate (MIR) per 1000 mosquitoes tested was calculated for each species infected by LACV (Table 2). MIRs ranged from 2.05–6.78/1000 for *Ae. triseriatus* depending on the survey and year of sampling and were about 10-fold higher than those estimated for *Ae. cinereus* (0.24 and 0.33/1000). LACV was also isolated from single pools of *Ae. canadensis* and *Ae. trivittatus* from the Hamden site in 2018 yielding MIRs of 1.18 and 1.09/1000, respectively. The total number of mosquitoes collected and tested by species are given in Additional file 2: Table S2.

**Table 2:** LACV minimal infection rates (MIR) per 1000 mosquitoes tested

Survey	Location	Year	Species	Total collected	Pools tested	LACV isolates	MIR (95% CI)
#1 Surveillance programme	Fairfield county	2005	*Ae. triseriatus*	340	163	1	2.94 (0.00–8.70)
		2015	*Ae. triseriatus*	487	186	1	2.05 (0.00–6.07)
		2016	*Ae. triseriatus*	319	158	1	3.13 (0.00–9.27)
		2018	*Ae. cinereus*	8469	472	2	0.24 (0.00–0.56)
#2 Lure study	Hamden	2018	*Ae. canadensis*	844	27	1	1.18 (0.00–3.51)
			*Ae. triseriatus*	713	32	2	2.81 (0.00–6.69)
			*Ae. trivittatus*	918	29	1	1.09 (0.00–3.22)
#3 La Crosse virus foci	Fairfield county	2018	*Ae. cinereus*	3029	218	1	0.33 (0.00–0.98)
			*Ae. triseriatus*	738	150	5	6.78 (0.86–12.69)

In addition, we collected *Aedes* eggs via ovitraps to assess vertical transmission of LACV to mosquito offspring. A total of 1204 *Ae. japonicus*, 13 *Aedes albopictus*, and 5818 *Ae. triseriatus* (both male and female) were collected at Easton, Redding and Fairfield sites and reared to adults. None of these F_1_ mosquitoes tested positive for LACV.

### Phylogenetic data

**Fig. 2:**
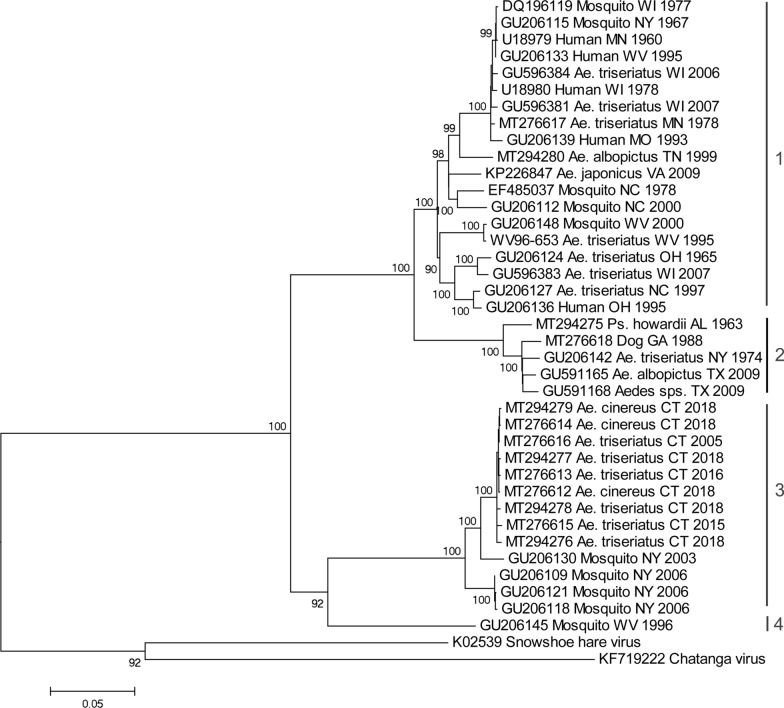
Phylogenetic comparison of novel LACV isolates from Connecticut, based on maximum likelihood analysis of the complete coding sequence of the M-segment polyprotein gene. Numbers at branch nodes indicate bootstrap support for 1000 replicates. Taxon labels specify the GenBank number, host, state, and year of collection

To evaluate the phylogenetic relationships of the Connecticut strains of LACV, we analyzed the entire coding sequence of the M segment polyprotein gene (4325 nt). Figure 2 shows the maximum likelihood tree for 40 viral sequences. LACV could be distinguished into a number of lineages as previously defined. Lineage 1 comprised strains from the Midwest and Appalachian regions but also included one earlier strain from New York (1967). Viruses from southeastern USA and one strain from New York (1974) grouped together as lineage 2. The Connecticut strains (2005–2018) shared 99.5–99.9% sequence identity to each other and formed a monophyletic clade that was most closely related to more recent strains from New York (2003–2006) to form lineage 3. In addition, a virus from West Virginia (1996) formed a fourth evolutionary lineage that was most closely related to the strains from lineage 3. The Connecticut strains of LACV were further characterized by analyzing an 883 nt portion of the S segment encoding the nucleocapsid and non-structural S protein and a 1037 nt portion of the L segment RNA-dependent RNA polymerase gene. These sequences were 99.6–100% similar to each other and most similar (97.0–98.4%) to the New York (2003–2006) strains of LACV. Novel sequences of the S, M and L segments of LACV isolates mentioned in this study were deposited in GenBank. Accession numbers for reference or novel sequences are listed in Additional file 1: Table S1.

## Discussion

La Crosse virus (LACV) is a member of the California serogroup viruses, which include Jamestown Canyon virus, California encephalitis virus, and snowshoe hare virus. LACV is most commonly associated with the Midwest and Appalachian regions of the USA. No locally acquired clinical cases have been documented in New England, and only one human case reported in New York State a decade ago. However, we show that through intensive targeted trapping, further evidence of LACV activity in mosquitoes in the Northeast is produced. Following the original detection in 2005, no isolates of LACV were obtained in Connecticut for 10 further years. Corroborating data from a focused-surveillance studies with routine monitoring data in Connecticut, and isolates from previous years (2005, 2015 and 2016) enabled evidence of localized persistence of novel regional variants of LACV. Twelve of the fourteen new isolates were detected in 2018, when species-directed sampling efforts were increased to assess the persistence of this virus. To be noted, 2018 was a year when higher than usual numbers of mosquitoes, as well as arbovirus detections, were observed in general under the standard routine sampling in Connecticut (our unpublished data, [14]), and this increased catch size may also have contributed to our detection of LACV.

*Aedes triseriatus* has been implicated as the main vector involved in the maintenance and transmission of LACV [15]. LACV isolates described here were detected between July and October from four different mosquito species but were most frequently detected in *Ae. triseriatus*, providing further evidence to support its role as the primary vector. Our detection of LACV in *Ae. cinereus* and *Ae. trivittatus* represents a novel finding in the USA. *Aedes cinereus* is frequently captured in Connecticut, and a source of several other arbovirus isolations as well, including Cache Valley, eastern equine encephalitis, Jamestown Canyon, and West Nile viruses [11]. Nevertheless, detection of LACV in a particular mosquito species does not necessarily incriminate it as a competent vector for the virus, and further studies are needed to elucidate its respective role in LACV transmission in the region. Outside of the northeastern USA, LACV has been isolated from a number of mammalian-biting mosquito species in addition to *Ae. triseriatus*, including *Ae. albopictus*, *Ae. canadensis*, *Ae. japonicus*, *Ae. vexans* and *Psorophora howardii* [6, 16–20]. *Aedes albopictus* and *Ae. japonicus* are invasive mosquito species emerging in Connecticut [21, 22]; however, no LACV isolates were found from either of these species in this study. Our detection of LACV in a pool of *Ae. canadensis* reinforces findings in Ohio which showed this species to be a secondary vector of LACV, via both field isolations of the virus and vector competence studies [23]; *Ae. vexans* is also thought to be an accessory vector for LACV in Virginia [24]. There is also experimental evidence that *Culex* spp. may play a limited role in LACV transmission dynamics [25].

Sciurids (chipmunks and squirrels) serve as the main vertebrate hosts for horizontal transmission and amplification of LACV, and a high rate of transovarial transmission is also reported in *Ae. triseriatus* in other regions of the USA [26, 27]. The contribution of both vertical and horizontal transmission requires further study to better understand LACV dynamics in the northeastern USA. Our study here included limited testing for evidence of vertical transmission in F1 mosquitoes reared from field-collected eggs, none of which tested positive. Finding no evidence of LACV in F_1_ mosquitoes could be surprising, given the high rate of vertical transmission reported elsewhere in the USA. For example, minimal infection rates of LACV in mosquitoes collected as eggs ranged from 0.4 to 7.5/1000 in study sites in West Virginia [28]. However, greatly increased numbers of field samples are required, as well as controlled experiments in the laboratory, to fully assess the role of vertical transmission. Reduced levels of vertical transmission of virus in mosquitoes could be one reason why LACV is infrequently detected in the northeastern USA. Miller et al. [27] report that LACV remained infective to vertebrate hosts after eight transovarial passages in *Ae. triseriatus* with infection rates of up to 71% in the offspring of an infected female. From this, they estimated that LACV can persist four years or longer in the vector population in the absence of horizontal amplification in vertebrate hosts. Transovarial transmission (TOT) is ecologically significant in the persistence of many vector-borne pathogens and used frequently by members of *Bunyavirales* when classical horizontal transmission is not possible [29].

MIRs can be seen to be high in 2015 and 2016, reflecting that the small quantity of *Ae. triseriatus* captured also yielded LACV. Our focused study in 2017–2018 aimed to improve the catch rate of this mosquito species to examine LACV in the region. Routine mosquito surveillance in Connecticut and throughout much of the northeast, largely focuses on the use of CDC light and gravid traps. Few adult *Ae. triseriatus* were captured during routine mosquito surveillance using CDC light traps, gravid traps, or standard deployment of BG-Sentinel traps, and it was not until our focused-LACV study 2017–2018 using a wide variety of different traps, that we saw numbers of this species increase. We suggest BG-Sentinel baited not only with BG-lure, but additionally with CO_2_, may enable increased collections of adult *Ae. triseriatus*, and thus enhanced chance of detecting LACV if it is present in an area [3]; once commercially available, additional baits described Eastwood et al. [10] are even more effective than BG-lure. Minimal field infection rates reported elsewhere have ranged from 0.26 to 27 per 1000 mosquitoes tested [15, 28, 30, 31].

Variants of LACV identified during this study represent a distinct third lineage. In other geographical regions of the USA, where lineages I and II occur, the virus is associated with clinical symptoms of human disease. Conversely, to our knowledge, locally acquired clinical cases of La Crosse encephalitis are exceedingly rare in the northeastern USA. Whether this is due to under-diagnosis of clinical illness to LACV (lack of case recognition), differences in virus strain virulence, a low prevalence of infection in mosquito vectors that effectively limits human exposure to biting activity in regions where the virus circulates, or limited vector competence by these mosquito species, remains to be determined. Clearly, given the presence of this arbovirus in mosquitoes in several areas of CT, there appears to exist an entomological risk. Furthermore, this risk occurs where there is currently no reported human disease, yet a sizeable human population which could come into contact with LACV-infected mosquitoes. Human serosurveys, in regions where the novel lineage of virus circulates, would be valuable to determining exposure rates and further illuminating epidemiology or possible differences in virulence.

Our findings clearly warrant that further investigation be taken to assess the public health risk that LACV (lineage-III) may pose to this region of the USA. There is a requirement for (i) vector competency studies with local populations of *Ae. triseriatus* and other identified mosquito species; (ii) infection and virulence studies in an animal model; (iii) assessment of the role of enzootic vertebrate reservoir hosts for this lineage of LACV, and (iv) vertical transmission studies in *Ae. triseriatus*.

## Conclusions

In conclusion, there is a low-level prevalence of lineage-III LACV in Connecticut in unique local populations maintained independently of known areas of LACV with reported clinical cases, i.e. Midwest and Appalachia regions. We have identified two new mosquito species (*Ae. cinereus* and *Ae. trivittatus*) that acquire LACV infection and may be involved in virus transmission in addition to the natural vector *Ae. triseriatus*. Greater awareness is needed to assess and highlight the potential health risk of lineage-III strains of LACV in the northeastern USA.

## Supplementary information

**Additional file 1: Table S1.** La Crosse virus sequences—GenBank accession numbers.

**Additional file 2: Table S2.** Mosquito species detected during the study, by survey and year, indicating the source of LACv isolates and the minimum infection rates (95% confidence limits).

## Data Availability

All data generated or analyzed during this study are included in this published article and its additional files.

## Abbreviations

CDC: Centers for Disease Control & Prevention (USA)
CPE: cytopathic effect
LACV: La Crosse virus
MIR: minimum infection rate
RNA: ribonucleic acid
RT-PCR: reverse-transcription polymerase chain reaction
TOT: transovarial transmission
