# Supplementary material for: Local persistence of novel regional variants of La Crosse virus in the Northeast USA
Source: Parasit Vectors. 2020 Nov 11;13:569. doi: 10.1186/s13071-020-04440-4 (PMC7659055; doi:10.1186/s13071-020-04440-4)
Supplement: Supplementary file 2 — Additional file 2: Table S2. Mosquito species detected during the study, by survey and year, indicating the source of LACv isolates and the minimum infection rates (95% confidence limits). [file 13071_2020_4440_MOESM2_ESM.docx]

| **Survey** | **Year** | **Species** | **Total Collected** | **LACv Isolates** | **MIR (95% Conf. Limit)** |
| --- | --- | --- | --- | --- | --- |
| #1 Surveillance Program- Fairfield County | 2005 | *Aedes abserratus* | 223 |  |  |
|  |  | *Aedes aurifer* | 1290 |  |  |
|  |  | *Aedes canadensis* | 2998 |  |  |
|  |  | *Aedes cantator* | 1636 |  |  |
|  |  | *Aedes cinereus* | 2456 |  |  |
|  |  | *Aedes communis* | 1 |  |  |
|  |  | *Aedes excrucians* | 103 |  |  |
|  |  | *Aedes japonicus* | 735 |  |  |
|  |  | *Aedes sollicitans* | 2533 |  |  |
|  |  | *Aedes sticticus* | 500 |  |  |
|  |  | *Aedes stimulans* | 161 |  |  |
|  |  | *Aedes taeniorhynchus* | 4745 |  |  |
|  |  | *Aedes thibaulti* | 1751 |  |  |
|  |  | *Aedes triseriatus* | 340 | 1 | 2.94 (0.00 - 8.70) |
|  |  | *Aedes trivittatus* | 280 |  |  |
|  |  | *Aedes vexans* | 4359 |  |  |
|  |  | *Anopheles barberi* | 4 |  |  |
|  |  | *Anopheles punctipennis* | 515 |  |  |
|  |  | *Anopheles quadrimaculatus* | 119 |  |  |
|  |  | *Anopheles walkeri* | 220 |  |  |
|  |  | *Coquillettidia perturbans* | 2113 |  |  |
|  |  | *Culex pipiens* | 5390 |  |  |
|  |  | *Culex restuans* | 1936 |  |  |
|  |  | *Culex salinarius* | 1276 |  |  |
|  |  | *Culex territans* | 10 |  |  |
|  |  | *Culiseta melanura* | 2264 |  |  |
|  |  | *Culiseta minnesotae* | 2 |  |  |
|  |  | *Culiseta morsitans* | 21 |  |  |
|  |  | *Orthopodomyia signifera* | 3 |  |  |
|  |  | *Psorophora ferox* | 361 |  |  |
|  |  | *Uranotaenia sapphirina* | 608 |  |  |
|  |  |  |  |  |  |
| #1 Surveillance Program- Fairfield County | 2015 | *Aedes abserratus* | 193 |  |  |
|  |  | *Aedes albopictus* | 214 |  |  |
|  |  | *Aedes aurifer* | 258 |  |  |
|  |  | *Aedes canadensis* | 8494 |  |  |
|  |  | *Aedes cantator* | 682 |  |  |
|  |  | *Aedes cinereus* | 1640 |  |  |
|  |  | *Aedes excrucians* | 123 |  |  |
|  |  | *Aedes hendersoni* | 1 |  |  |
|  |  | *Aedes japonicus* | 965 |  |  |
|  |  | *Aedes sollicitans* | 402 |  |  |
|  |  | *Aedes sticticus* | 34 |  |  |
|  |  | *Aedes stimulans* | 52 |  |  |
|  |  | *Aedes taeniorhynchus* | 11407 |  |  |
|  |  | *Aedes thibaulti* | 2041 |  |  |
|  |  | *Aedes triseriatus* | 487 | 1 | 2.05 (0.00 – 6.07) |
|  |  | *Aedes trivittatus* | 849 |  |  |
|  |  | *Aedes vexans* | 3329 |  |  |
|  |  | *Anopheles barberi* | 3 |  |  |
|  |  | *Anopheles crucians* | 2 |  |  |
|  |  | *Anopheles punctipennis* | 838 |  |  |
|  |  | *Anopheles quadrimaculatus* | 363 |  |  |
|  |  | *Anopheles walkeri* | 1304 |  |  |
|  |  | *Coquillettidia perturbans* | 4035 |  |  |
|  |  | *Culex erraticus* | 15 |  |  |
|  |  | *Culex pipiens* | 6013 |  |  |
|  |  | *Culex restuans* | 1537 |  |  |
|  |  | *Culex salinarius* | 4274 |  |  |
|  |  | *Culex territans* | 96 |  |  |
|  |  | *Culiseta melanura* | 792 |  |  |
|  |  | *Culiseta minnesotae* | 1 |  |  |
|  |  | *Culiseta morsitans* | 8 |  |  |
|  |  | *Orthopodomyia signifera* | 3 |  |  |
|  |  | *Psorophora columbiae* | 1 |  |  |
|  |  | *Psorophora ferox* | 672 |  |  |
|  |  | *Uranotaenia sapphirina* | 1048 |  |  |
|  |  |  |  |  |  |
| #1 Surveillance Program- Fairfield County | 2016 | *Aedes abserratus* | 293 |  |  |
|  |  | *Aedes albopictus* | 2119 |  |  |
|  |  | *Aedes cinereus* | 1072 |  |  |
|  |  | *Aedes vexans* | 2493 |  |  |
|  |  | *Anopheles barberi* | 6 |  |  |
|  |  | *Anopheles crucians* | 16 |  |  |
|  |  | *Anopheles punctipennis* | 315 |  |  |
|  |  | *Anopheles quadrimaculatus* | 169 |  |  |
|  |  | *Anopheles walkeri* | 325 |  |  |
|  |  | *Coquillettidia perturbans* | 4531 |  |  |
|  |  | *Culex erraticus* | 248 |  |  |
|  |  | *Culex pipiens* | 16691 |  |  |
|  |  | *Culex restuans* | 2761 |  |  |
|  |  | *Culex salinarius* | 4517 |  |  |
|  |  | *Culex territans* | 28 |  |  |
|  |  | *Culiseta melanura* | 421 |  |  |
|  |  | *Culiseta morsitans* | 17 |  |  |
|  |  | *Aedes abserratus* | 293 |  |  |
|  |  | *Aedes atlanticus* | 2 |  |  |
|  |  | *Aedes aurifer* | 940 |  |  |
|  |  | *Aedes canadensis* | 4793 |  |  |
|  |  | *Aedes cantator* | 133 |  |  |
|  |  | *Aedes excrucians* | 57 |  |  |
|  |  | *Aedes grossbecki* | 3 |  |  |
|  |  | *Aedes japonicus* | 774 |  |  |
|  |  | *Aedes sollicitans* | 384 |  |  |
|  |  | *Aedes stimulans* | 88 |  |  |
|  |  | *Aedes taeniorhynchus* | 14261 |  |  |
|  |  | *Aedes thibaulti* | 2149 |  |  |
|  |  | *Aedes triseriatus* | 319 | 1 | 3.13 (0.00 – 9.27) |
|  |  | *Aedes trivittatus* | 490 |  |  |
|  |  | *Orthopodomyia signifera* | 7 |  |  |
|  |  | *Psorophora columbiae* | 1 |  |  |
|  |  | *Psorophora ferox* | 455 |  |  |
|  |  | *Psorophora howardii* | 6 |  |  |
|  |  | *Uranotaenia sapphirina* | 503 |  |  |
|  |  |  |  |  |  |
| #1 Surveillance Program- Fairfield County | 2018 | *Aedes abserratus* | 418 |  |  |
|  |  | *Aedes albopictus* | 2298 |  |  |
|  |  | *Aedes atlanticus* | 14 |  |  |
|  |  | *Aedes atropalpus* | 1 |  |  |
|  |  | *Aedes aurifer* | 1256 |  |  |
|  |  | *Aedes canadensis* | 16142 |  |  |
|  |  | *Aedes cantator* | 427 |  |  |
|  |  | *Aedes cinereus* | 8469 | 2 | 0.24 (0.00 – 0.56) |
|  |  | *Aedes excrucians* | 681 |  |  |
|  |  | *Aedes grossbecki* | 19 |  |  |
|  |  | *Aedes infirmatus* | 9 |  |  |
|  |  | *Aedes japonicus* | 1144 |  |  |
|  |  | *Aedes sollicitans* | 272 |  |  |
|  |  | *Aedes sticticus* | 100 |  |  |
|  |  | *Aedes stimulans* | 219 |  |  |
|  |  | *Aedes taeniorhynchus* | 12069 |  |  |
|  |  | *Aedes thibaulti* | 8768 |  |  |
|  |  | *Aedes triseriatus* | 753 |  |  |
|  |  | *Aedes trivittatus* | 620 |  |  |
|  |  | *Aedes vexans* | 12530 |  |  |
|  |  | *Anopheles barberi* | 3 |  |  |
|  |  | *Anopheles crucians* | 23 |  |  |
|  |  | *Anopheles punctipennis* | 1901 |  |  |
|  |  | *Anopheles quadrimaculatus* | 171 |  |  |
|  |  | *Anopheles walkeri* | 1183 |  |  |
|  |  | *Coquillettidia perturbans* | 18992 |  |  |
|  |  | *Culex erraticus* | 196 |  |  |
|  |  | *Culex pipiens* | 16929 |  |  |
|  |  | *Culex restuans* | 5993 |  |  |
|  |  | *Culex salinarius* | 7836 |  |  |
|  |  | *Culex territans* | 96 |  |  |
|  |  | *Culiseta melanura* | 7170 |  |  |
|  |  | *Culiseta minnesotae* | 2 |  |  |
|  |  | *Culiseta morsitans* | 203 |  |  |
|  |  | *Orthopodomyia signifera* | 2 |  |  |
|  |  | *Psorophora columbiae* | 10 |  |  |
|  |  | *Psorophora ferox* | 9824 |  |  |
|  |  | *Psorophora howardii* | 39 |  |  |
|  |  | *Toxorhynchites r. septentrionalis* | 2 |  |  |
|  |  | *Uranotaenia sapphirina* | 910 |  |  |
|  |  |  |  |  |  |
| #2 Lure Study – Hamden | 2018 | *Aedes abserratus* | 1 |  |  |
|  |  | *Aedes albopictus* | 6 |  |  |
|  |  | *Aedes canadensis* | 844 | 1 | 1.18 (0.00 - 3.51) |
|  |  | *Aedes cantator* | 34 |  |  |
|  |  | *Aedes cinereus* | 16 |  |  |
|  |  | *Aedes excrucians* | 6 |  |  |
|  |  | *Aedes hendersonii* | 3 |  |  |
|  |  | *Aedes japonicus* | 4552 |  |  |
|  |  | *Aedes sollicitans* | 10 |  |  |
|  |  | *Aedes sticticus* | 6 |  |  |
|  |  | *Aedes stimulans* | 81 |  |  |
|  |  | *Aedes taeniorhynchus* | 5 |  |  |
|  |  | *Aedes triseriatus* | 713 | 2 | 2.81 (0.00 - 6.69) |
|  |  | *Aedes trivittatus* | 918 | 1 | 1.09 (0.00 – 3.22) |
|  |  | *Aedes vexans* | 1457 |  |  |
|  |  | *Anopheles barberi* | 4 |  |  |
|  |  | *Anopheles punctipennis* | 3981 |  |  |
|  |  | *Anopheles quadrimaculatus* | 402 |  |  |
|  |  | *Anopheles walkeri* | 3 |  |  |
|  |  | *Coquillettidia perturbans* | 271 |  |  |
|  |  | *Culex erraticus* | 2 |  |  |
|  |  | *Culex pipiens* | 75 |  |  |
|  |  | *Culex restuans* | 3 |  |  |
|  |  | *Culex salinarius* | 384 |  |  |
|  |  | *Psorophora columbiae* | 3 |  |  |
|  |  | *Psorophora ferox* | 99 |  |  |
|  |  |  |  |  |  |
| #3 La Crosse Virus Foci Fairfield County | 2017 | *Aedes albopictus* | 3 |  |  |
|  |  | *Aedes canadensis* | 275 |  |  |
|  |  | *Aedes cinereus* | 224 |  |  |
|  |  | *Aedes japonicus* | 25 |  |  |
|  |  | *Aedes sollicitans* | 1 |  |  |
|  |  | *Aedes thibaulti* | 154 |  |  |
|  |  | *Aedes triseriatus* | 130 |  |  |
|  |  | *Aedes trivittatus* | 4 |  |  |
|  |  | *Aedes vexans* | 55 |  |  |
|  |  | *Anopheles barberi* | 1 |  |  |
|  |  | *Anopheles punctipennis* | 112 |  |  |
|  |  | *Anopheles quadrimaculatus* | 1 |  |  |
|  |  | *Anopheles walkeri* | 1 |  |  |
|  |  | *Coquillettidia perturbans* | 24 |  |  |
|  |  | *Culex erraticus* | 9 |  |  |
|  |  | *Culex pipiens* | 12 |  |  |
|  |  | *Culex restuans* | 22 |  |  |
|  |  | *Culex salinarius* | 57 |  |  |
|  |  | *Culex territans* | 5 |  |  |
|  |  | *Culiseta melanura* | 33 |  |  |
|  |  | *Culiseta morsitans* | 3 |  |  |
|  |  | *Psorophora ferox* | 3 |  |  |
|  |  | *Uranotaenia sapphirina* | 169 |  |  |
|  |  |  |  |  |  |
| #3 La Crosse Virus Foci Fairfield County | 2018 | *Aedes abserratus* | 269 |  |  |
|  |  | *Aedes aurifer* | 51 |  |  |
|  |  | *Aedes canadensis* | 8796 |  |  |
|  |  | *Aedes cinereus* | 3029 | 1 | 0.33 (0.00 – 0.98) |
|  |  | *Aedes excrucians* | 140 |  |  |
|  |  | *Aedes grossbecki* | 2 |  |  |
|  |  | *Aedes infirmatus* | 1 |  |  |
|  |  | *Aedes japonicus* | 175 |  |  |
|  |  | *Aedes sticticus* | 63 |  |  |
|  |  | *Aedes stimulans* | 36 |  |  |
|  |  | *Aedes thibaulti* | 3382 |  |  |
|  |  | *Aedes triseriatus* | 738 | 5 | 6.78 (0.86-12.69) |
|  |  | *Aedes trivittatus* | 60 |  |  |
|  |  | *Aedes vexans* | 380 |  |  |
|  |  | *Anopheles barberi* | 6 |  |  |
|  |  | *Anopheles punctipennis* | 2119 |  |  |
|  |  | *Anopheles quadrimaculatus* | 7 |  |  |
|  |  | *Anopheles walkeri* | 17 |  |  |
|  |  | *Coquillettidia perturbans* | 150 |  |  |
|  |  | *Culex erraticus* | 5 |  |  |
|  |  | *Culex pipiens* | 36 |  |  |
|  |  | *Culex restuans* | 189 |  |  |
|  |  | *Culex salinarius* | 101 |  |  |
|  |  | *Culex territans* | 4 |  |  |
|  |  | *Culiseta melanura* | 59 |  |  |
|  |  | *Culiseta morsitans* | 12 |  |  |
|  |  | *Psorophora ferox* | 247 |  |  |
|  |  | *Psorophora howardii* | 1 |  |  |
|  |  | *Uranotaenia sapphirina* | 211 |  |  |
